# Mutualistic interactions between ants and fungi: A review

**DOI:** 10.1002/ece3.10386

**Published:** 2023-07-28

**Authors:** Alain Dejean, Frédéric Azémar, Piotr Naskrecki, Maurice Tindo, Vivien Rossi, Christian Faucher, Hervé Gryta

**Affiliations:** ^1^ Laboratoire Écologie Fonctionnelle et Environnement Université de Toulouse, CNRS, Toulouse INP, Université Toulouse 3 – Paul Sabatier (UPS) Toulouse France; ^2^ UMR EcoFoG, AgroParisTech Cirad, CNRS, INRA, Université des Antilles, Université de Guyane Kourou France; ^3^ Museum of Comparative Zoology Harvard University Cambridge Massachusetts USA; ^4^ Laboratory of Biology and Physiology of Animal Organisms, Faculty of Science University of Douala Douala Cameroon; ^5^ Remote Sensing and Forest Ecology Lab, Higher Teacher's Training College Marien Ngouabi University Brazzaville Democratic Republic of the Congo; ^6^ R U Forests and Societies, CIRAD Brazzaville Democratic Republic of the Congo; ^7^ Laboratoire Evolution & Diversité Biologique (EDB UMR 5174) CNRS IRD, Université Toulouse 3 Toulouse France

**Keywords:** ant constructions, ant fungiculture, composite materials, defoliation, fungi as food

## Abstract

The large amount of dead plant biomass caused by the final extinction events triggered a fungi proliferation that mostly differentiated into saprophytes degrading organic matter; others became parasites, predators, likely commensals, and mutualists. Among the last, many have relationships with ants, the most emblematic seen in the Neotropical myrmicine Attina that cultivate Basidiomycota for food. Among them, leaf‐cutting, fungus‐growing species illustrate an ecological innovation because they grow fungal gardens from fresh plant material rather than arthropod frass and plant debris. Myrmecophytes shelter “plant‐ants” in hollow structures, the domatia, whose inner walls are lined with thin‐walled Ascomycota hyphae that, in certain cases, are eaten by the ants, showing a form of convergence. Typically, these Ascomycota have antibacterial properties illustrating cases of farming for protection. Ant gardens, or mutualistic associations between certain ant species and epiphytes, shelter endophytic fungi that promote the growth of the epiphytes. Because the cell walls of certain Ascomycota hyphae remain sturdy after the death of the mycelium, they form resistant fibers used by ants to reinforce their constructions (e.g., galleries, shelters for tended hemipterans, and carton nests). Thus, we saw cases of “true” fungal agriculture involving planting, cultivating, and harvesting Basidiomycota for food with Attina. A convergence with “plant‐ants” feeding on Ascomycota whose antibacterial activity is generally exploited (i.e., farming for protection). The growth of epiphytes was promoted by endophytic fungi in ant gardens. Finally, farming for structural materials occurred with, in one case, a leaf‐cutting, fungus‐growing ant using Ascomycota fibers to reinforce its nests.

## Graphical abstract

We review the mutualistic interactions between ants and fungi. Neotropical myrmicine Attina cultivate Basidiomycota for food, the same being true for certain “plant‐ants” associated with myrmecophytes that also feed on Ascomycota fungi. Otherwise, these Ascomycota have antibacterial properties. Ant gardens (mutualistic associations between ants and epiphytes) also shelter endophytic fungi that promote the growth of the epiphytes. Finally, the cell walls of Chaetothyriales and Capnodiales hyphae (both Ascomycota) remain sturdy after the death of the mycelium, forming resistant fibers used by ants to reinforce their constructions.

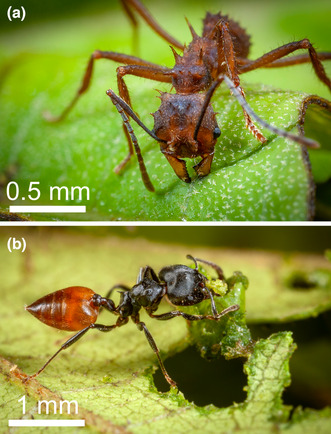

## 1 INTRODUCTION

For terrestrial environments, the Permian–Triassic extinction event (251.9 Mya) and the Cretaceous–Paleogene mass extinction (≈66 Mya) resulted in massive amounts of dead plant biomass, followed by the proliferation of fungi that then influenced the evolution of all other living terrestrial organisms (Berry, 2020; Heitman et al., 2018; Rampino & Eshet, 2018). Many fungi participate in the degradation of organic matter as saprophytes, whereas other taxa are parasites (e.g., entomopathogenic fungi), predators (e.g., of nematodes), likely commensals (e.g., yeasts from the guts of insects), and mutualists of chlorophyllous plants (e.g., algae in lichens; plants in mycorrhizal symbiosis) and animals (e.g., fungi cultivated for food by certain termites, coleopterans, and ants; Heitman et al., 2018; see also Biedermann & Vega, 2020 for mutualisms).

Ants, the focal taxon of this study, appeared around the Jurassic–Cretaceous boundary (≈145 Mya; Romiguier et al., 2022) and differentiated after the Cretaceous–Paleogene extinction event during the periods of proliferation of fungi, which explains why these two taxa evolved relationships ranging from parasitism to mutualisms (Heitman et al., 2018). Furthermore, ant diversification tracks the rise of angiosperms (Moreau et al., 2006).

In mutualisms, vertical transmission occurs when hosts transmit symbionts to their offspring, whereas in horizontal transmission the association needs to be re‐established every generation, but these associations are generally stable thanks to reciprocal by‐product benefits (Leeks et al., 2019; Leimar & Hammerstein, 2010). For instance, during the vertical transmission of associated fungi, the infrabuccal pockets that filter out solid particles permit founding queens to transport fungal hyphae and spores from their mother colony to their new incipient colony. In horizontal transmission, the mutualistic fungi can be found in nature or can originate from neighboring nests (Biedermann & Vega, 2020).

Here, we attempt to review all known cases of ants forming mutualistic associations with fungi (e.g., as food or an antibacterial agent or to reinforce their constructions) recorded to date.

## 2 ATTINA ANTS USING FUNGI AS FOOD

**FIGURE 1:**
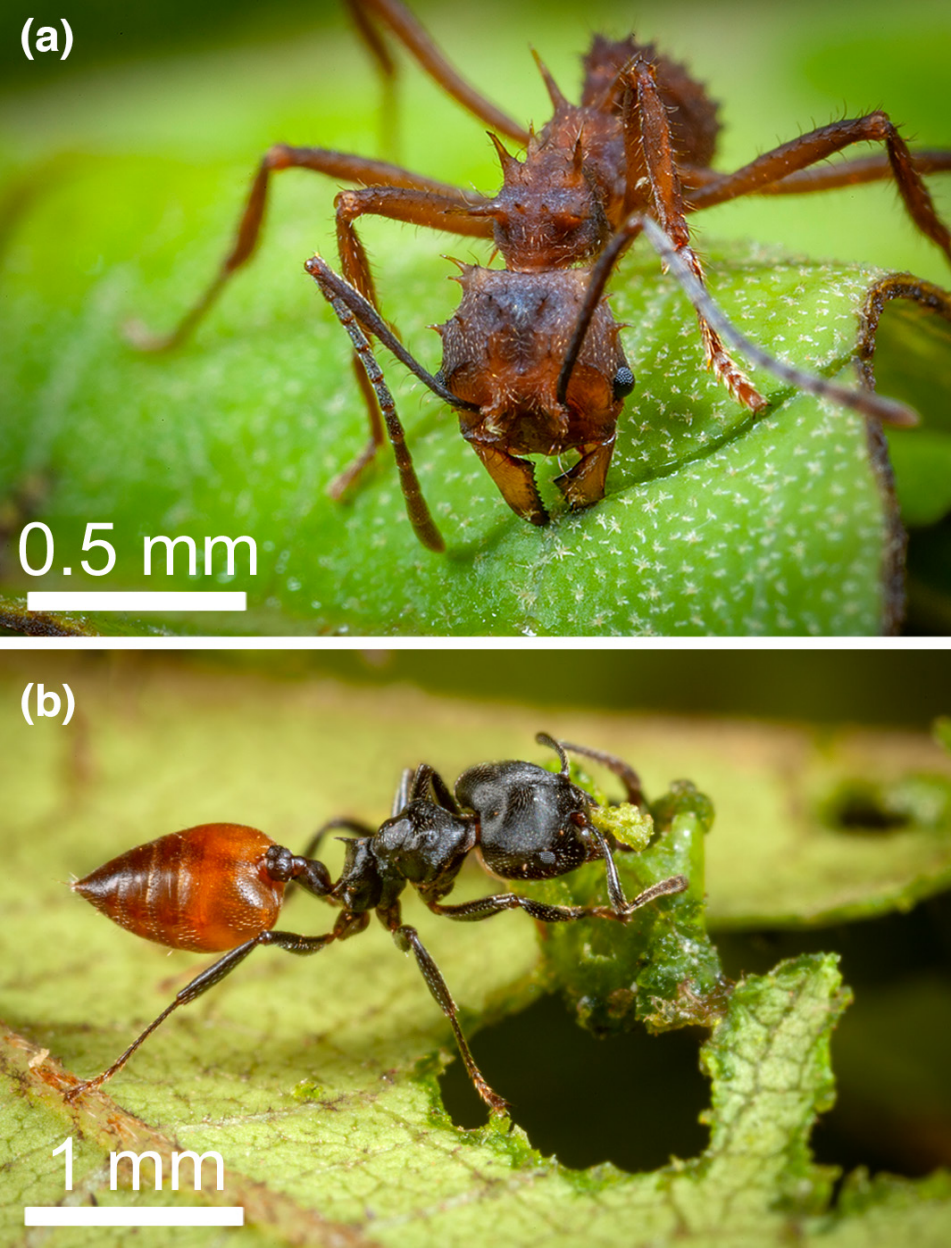
(a) *Acromyrmex* sp. worker positions its hind legs on the leaf edge and rotates around them while cutting an arc in a leaf blade, whereas it uses its mandibles asymmetrically, one leading the process, the other cutting a large piece of leaf. (b) By comparison, a *Crematogaster clariventris* worker cuts small pieces with a typical chewing motion of its mandibles moving symmetrically (photos Piotr Naskrecki).

The most emblematic case of an ant‐fungi mutualism is the food‐based relationship involving the Attina New World subtribe (subfamily Myrmicinae; tribe Attini) that appeared 61–57 Mya during the recovery period that followed the Cretaceous–Paleogene mass extinction (Branstetter et al., 2017). The Attina, which cultivate basidiomycete fungi of the order Agaricales in an obligate association, comprise 19 genera that developed five modes of fungal agriculture as they planted, cultivated, and harvested fungi (Mueller et al., 2005; Schultz, 2021). (1) The lower Attina domesticated cultivars from free‐living Leucocoprineae in a facultative mutualism. Two exceptions exist among the lower Attina. *Apterostigma megacephala* cultivates the most derived higher attine cultivar, *Leucoagaricus gongylophorus*, but, like all other lower Attina, it employs a garden substrate consisting mostly of arthropod frass and plant debris (also used by non‐leaf‐cutting higher Attina). Indeed, the workers collect leaflets of a Fabaceae to line the floor of their garden chamber; these leaflets are not incorporated into the fungal garden. In contrast, *Paramycetophylax bruchi* workers collect fresh leaflets from another Fabaceae, *Prosopis flexuosa*, to serve as a substrate for their fungal cultivar, something suggesting leaf‐cutting, fungus‐growing behavior, the leaflets being gathered whole. (2) Coral fungus agriculture is limited to the *Apterostigma pilosum* group that cultivates two clades of Pterulaceae, but it is unknown whether they are free‐living or not. (3) Yeast agriculture was described for *Cyphomyrmex* (belongs to the higher Attina) species that cultivate unicellular leucocoprineaceous with free‐living conspecifics, so that the mutualism is likely facultative. (4) The higher Attina domesticated two derived, polyploid leucocoprineaceous cultivar clades incapable of surviving on their own (obligate mutualism); they produce hyphal tip swellings, the gongylidia, specifically to feed them. These “non‐leaf‐cutting” species collect as a substrate for their cultivars arthropod feces, plant detritus, insect corpses, and fresh plant parts (e.g., seeds, flowers, and fruit pulp); *Trachymyrmex* and *Sericomyrmex* can exceptionally harvest fresh leaves and flowers (Ronque et al., 2019). (5) Among the higher Attina, leaf‐cutting behavior appeared 18–19 Mya (Myocene: 23.03–5.33 Mya), an ecological innovation involving the genera *Atta*, *Acromyrmex*, and *Amoimyrmex*. These leaf‐cutting taxa, which have an obligatory association mostly with *Leucoagaricus gongylophorus* (Agaricaceae), are considered the most evolved Attina. Indeed, compared with small, monomorphic colonies of non‐leaf‐cutting Attina with simple nests (e.g., one or a few chambers), leaf‐cutting ants have populated nests with many chambers, and a worker polyphenism associated with a fine‐tuned division of labor. Overall, they use fresh plant material as a substrate to grow fungal gardens (Barrera et al., 2022; Bizarria Jr. et al., 2022; Branstetter et al., 2017; Cristiano et al., 2020; Hanisch et al., 2022; Mueller et al., 2018; Schultz, 2021; Sosa‐Calvo et al., 2017). In Attina, “fungus‐growing” corresponds to what Mueller et al. (2005) called “true fungal agriculture” as this mode of cultivation is based on (1) planting, (2) cultivating, and (3) harvesting the crop for food in an obligate dependency (Figure 1) as opposed to farming for protection (e.g., antimicrobial activities) or structural materials (e.g., construction of nests or of a trap; Biedermann & Vega, 2020; see also Ivens, 2015).

The damage produced by leaf‐cutting Attina in Hispaniola was first cited by Las Casas (1559) who also succinctly described their nest. Indeed, leaf‐cutting ants are the principal Neotropical defoliators inflicting widespread forestial and agricultural damage due to their hyperabundance related to increasing levels of fragmentation, so that numerous approaches have been used to try to control them including satellite imagery and drones to estimate their presence in forests (Dionisi et al., 2021; Santos, Gonçalves Biesseck, et al., 2022; Santos, Santos, et al., 2022; Schowalter & Ring, 2017).

Overall, there is vertical transmission as, during the nuptial flight, young queens transport in their infrabuccal pockets pieces of their nest mycelium to start a fungal garden in their new colony. This does not preclude horizontal transfer, noted in the lower Attina and *Cyphomyrmex* (i.e., yeast agriculture), particularly during the founding stage when queens search for replacement cultivars and adopt orphaned gardens, including in a leaf‐cutting ant species (Bizarria Jr. et al., 2022; Branstetter et al., 2017; Howe et al., 2019).

Furthermore, Attina provision and maintain these gardens by removing potentially parasitic microfungi and eliminating parasites thanks to antifungals produced by a mixed community of actinomycete bacteria that grows on their cuticle. Among them, *Pseudonocardia* is first vertically transmitted by founding queens and then transferred from workers or even from the fungal cultivar to younger workers and new queens. Other actinomycetes, including *Streptomyces* spp., are horizontally acquired from the environment (Batey et al., 2020; Bruner‐Montero et al., 2021; Li et al., 2018).

## 3 ANTS OTHER THAN ATTINA USING FUNGI AS FOOD

Species of *Aphaenogaster*, *Pheidole*, *Tetramorium* (Myrmicinae), *Camponotus* (Formicinae), and *Iridomyrmex* (Dolichoderinae) occasionally feed on mushrooms (i.e., sporophores; Epps & Penick, 2018). But an exceptional adaptation to feeding on wild mushrooms has been recognized for Malaysian ants of the genus *Euprenolepis* (Formicinae) based on a stable trail network whose sections can be deactivated and reactivated, permitting the ants to detect mushrooms whose locations are rather predictable (Lizon à l'Allemand et al., 2019; von Beeren, Lizon à l'Allemand, et al., 2014; von Beeren, Mair, & Witte, 2014; Witte & Maschwitz, 2008).

Other cases of food‐based ant‐fungus associations concern ants feeding on fungal hyphae as do the Attina. Ants in the *Megalomyrmex silvestrii* group (Myrmicinae) are social parasites that can also usurp the nests of the Attina of the genera *Apterostigma*, *Cyphomyrmex*, *Trachymyrmex*, and *Sericomyrmex* (Adams, Mueller, Holloway, et al., 2000; Adams, Mueller, Schultz, & Norden, 2000; Mueller et al., 2001; Shik et al., 2018; Wheeler, 1925). They feed on the fungal gardens of their host colonies and can participate in tending the gardens by readjusting their overall size and shape, but not by cultivating fungi, for example, by adding substrate (Mueller et al., 2001). To parasitize *Sericomyrmex amabilis* colonies, *M. symmetochus* individuals use a chemical insignificance strategy (i.e., few cuticular hydrocarbons on their cuticle) and elicit submissive behavior from their host thanks to their alkaloid venom (Neupert et al., 2018). *Megalomyrmex symmetochus* also protect their host ants from raids by *Gnamptogenys hartmani* thanks to their efficacious volatile alkaloid venom. Indeed, in their absence, the *Sericomyrmex* colonies are exterminated. Also, the *Megalomyrmex* odor discourages the *Gnamptogenys* scouts from recruiting nestmates. When a *Gnamptogenys* worker survives an attack, it is then killed by its own nestmates due to a modification to its cuticular hydrocarbons likely coming from the volatile *Megalomyrmex* venom (Adams et al., 2013; see also Dejean et al. (2007) for *Pheidole* triggering attacks between army ants).

Other cases have been reported for plant‐ants or those ants associated with myrmecophytes (i.e., plants sheltering colonies of “plant‐ant” species in hollow structures called domatia, the ants protecting them from their enemies). Bailey (1920) intuited this when he noted that African and Neotropical pseudomyrmicine plant‐ants of the genera *Tetraponera* and *Pseudomyrmex* feed their larvae with pellets from their infrabuccal pocket that contain fungal spores and hyphae gathered from the mycelium lining their host myrmecophyte domatia. Indeed, three plant‐ant species gather the mycelia of Ascomycota of the order Chaetothyriales that grow in their host myrmecophyte domatia to feed their larvae. These include the African Formicinae *Petalomyrmex phylax* associated with *Leonardoxa africana* (Fabaceae) and two Pseudomyrmecinae, the African *Tetraponera aethiops* associated with *Barteria fistulosa* (Passifloraceae) and the Neotropical *Pseudomyrmex penetrator* associated with *Tachigali* sp. (Fabaceae; Blatrix et al., 2012; see also Defossez et al., 2009, 2011).

Four plant‐ants of the genus *Azteca* (Dolichoderinae), namely *A. alfari*, *A. coeruleipennis*, *A. constructor* and *A. xanthochroa*, associated with myrmecophytic *Cecropia* (Urticaceae) grow Chaetothyrialean hyphae in their host tree domatia (i.e., the internodes of the trees; Nepel et al., 2016). To find a new colony, each swarming queen, after being fecundated, needs to find a tree not already occupied by a well‐established *Azteca* colony, generally a young, <2‐m‐tall tree. Therefore, these queens can be easily observed digging a hole in a specific zone of an internode. These swarming queens carry in their infrabuccal pockets spores and hyphal fragments from their mother colony and begin to cultivate them after they are installed in an internode, illustrating a case of vertical transmission. There, they feed their first larvae with their cultivated fungal hyphae until the first worker appears and then feed all colony members mostly with food bodies, or Mullerian bodies, produced by the trichilia, or pads of clustered trichomes situated on the abaxial side of the leaf petioles of the host *Cecropia* (Mayer et al., 2018). Thus, as for the Attina, the queens of these plant‐ants transmit fungi vertically between parent and offspring nests (Blatrix et al., 2012; Mayer et al., 2018).

## 4 ANTIBACTERIAL ROLE OF ASSOCIATED FUNGI AND ELIMINATION OF PARASITIC FUNGI

One important role of mutualist fungal hyphae is related to their antibacterial properties first shown for the carton nests of the formicine ant *Lasius fuliginosus* (Vasse et al., 2017). Antibiotic compounds were also noted for the Chaetothyriales associated with myrmecophytes, permitting their domatia to remain free of pathogens (Moreno et al., 2019).

Furthermore, as for the Attina that eliminate fungal parasites thanks to the actinomycete bacteria that grow on their cuticle, *L. fuliginosus* inhibits a parasitic fungus, something shown using extracts of ant body parts (Brinker et al., 2019).

Farming for protection from bacteria was also noted for plant‐ants lining the inner walls of their host myrmecophyte domatia with fungal hyphae.

This was first noted for the Asian epiphytes *Myrmecodia* and *Hydnophyton* (Rubiaceae) associated with dolichoderine ants of the genera *Iridomyrmex* and *Philidris* (Chomicki et al., 2017; Huxley, 1978; Miehe, 1911; Volp & Lach, 2019), Chaetothyriales fungi being recorded as lining nursery and ventilation chambers (Greenfield et al., 2021). This was also noted for the phanerophyte *Endospermum formicarum* (Euphorbiaceae) associated with the formicine *Camponotus quadriceps* (Docters van Leeuwen, 1929). Among the phanerophytes, this was also noted for the genera *Barteria* (Passifloraceae), *Cecropia* (Urticaceae), *Cuviera* (Rubiaceae), *Enterolobium* (Fabaceae), *Nauclea* (Rubiaceae), *Plectronia* (Rubiaceae), *Sarcocephalus* (Rubiaceae), *Triplaris* (Polygonaceae), *Vachellia* (Fabaceae), and *Vitex* (Lamiaceae; Bailey, 1920).

**FIGURE 2:**
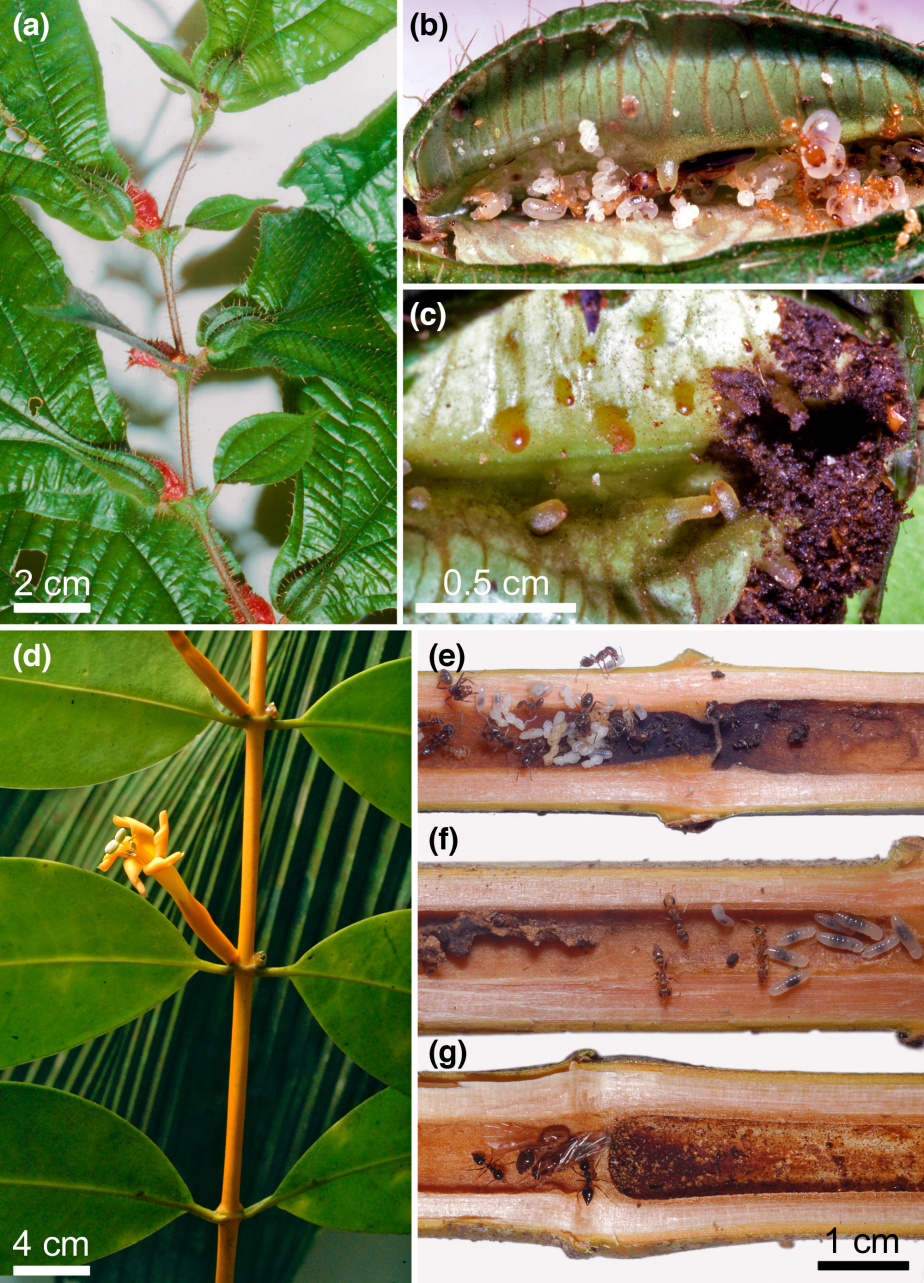
Myrmecophytes shelter certain ant species in hollow structures where the ants grow Ascomycota fungi likely for their antibacterial role. (a–c) *Maieta guianensis* (Melastomataceae) bears pairs of leaf pouches sheltering *Pheidole minutula* ants that nest in one pouch, while they deposit wastes in the other where fungi develop. (d) *Tachia guianensis* (Gentianaceae), here in bloom, can shelter in its hollow twigs several ant species that raise their brood close to or on their fungi‐rich deposits. (e) *Azteca* sp. (f) *Pseudomyrmex tenuis*. (g) *Crematogaster brasiliensis*. (f) (Alain Dejean, personal observation). Nutrient provisioning to the plant, or myrmecotrophy, was shown for both the *Maieta* and *Tachia* (Dejean et al., 2017; Solano & Dejean, 2004; photos Alain Dejean).

Defossez et al. (2009) also noted the presence of Chaetothyrialean hyphae lining the domatia of four other African myrmecophytes: *Calpocalyx cauliflorus* (Fabaceae) occupied by *Atopomyrmex calpocalycola* (Myrmicinae), *Kaetia hispida* (Rubiacea) occupied by *Crematogaster* sp., *Vitex grandifolia* (Lamiaceae) occupied by *Aphomomyrmex afer* (Formicinae), and *V. thyrsiflora* occupied by *Tetraponera tessmanni* (Pseudomyrmecinae). Furthermore, Vasse et al. (2017) reported cases from West African, SE Asian, and Neotropical myrmecophyte species where hyphae from several Chaetothyriales families are involved in different associations (see Figure 2 for Neotropical myrmecophytes). Indeed, Cyphellophoraceae were involved in the associations between *Pseudomyrmex* ants and myrmecophytic *Triplaris* or *Tachigali*, *Tetraponera* and *Barteria*, *Azteca* and *Cecropia*, *Cladomyrma* (Formicinae) and *Saraca* (Fabaceae) or *Crypteronia* (Crypteroniaceae), and *Crematogaster* and *Macaranga* (Euphorbiaceae). Also, closely related strains of hyphae from the Trichomeriaceae were noted for Neotropical *Azteca* ants associated with *Cecropia* and SE Asian *Cladomyrma* associated with *Saraca* (Fabaceae).

However, the fungal communities in the domatia of the East African myrmecophyte *Vachellia drepanolobium* include few Basidiomycota, whereas the Ascomycota of the classes Sordariomycetes, Saccharomycetes, and Dothideomycetes dominated, with among the Dothideomycetes a high proportion of the order Capnodiales (Baker et al., 2017).

The epiphytic SE Asian *Dischidia major* (Asclepiadaceae) shelters in its hollow leaves the nests of ants of the genus *Philidris* (Dolichoderinae). The inner surface of these leaves is lined with algal filaments and hyphae from five species of Chaetothyriales and four species of Capnodiales (Blatrix et al., 2021). In this mutualistic association, the host plant derives carbon from the ants' respiration and nitrogen from debris deposited by the workers in the leaf domatia (Treseder et al., 1995), but the role of the associated fungi in nutrient provisioning has not been demonstrated.

Ascomycota from the classes Eurotiomycetes (mostly of the order Chaetothyriales), Sordariomycetes, Saccharomycetes, and Dothideomycetes (mostly of the order Capnodiales) involved in lining the domatia of myrmecophytes, usually sporulating, have thin‐walled hyphae that form a dense layer on living host plant tissues. They are rather specific as, generally, there are only one or two fungal species per host myrmecophyte species and they differ from those involved in ant‐made constructions (see below; Vasse et al., 2017; Voglmayr et al., 2011).

## 5 FUNGAL HYPHAE INVOLVED IN ANT GARDENS

**FIGURE 3:**
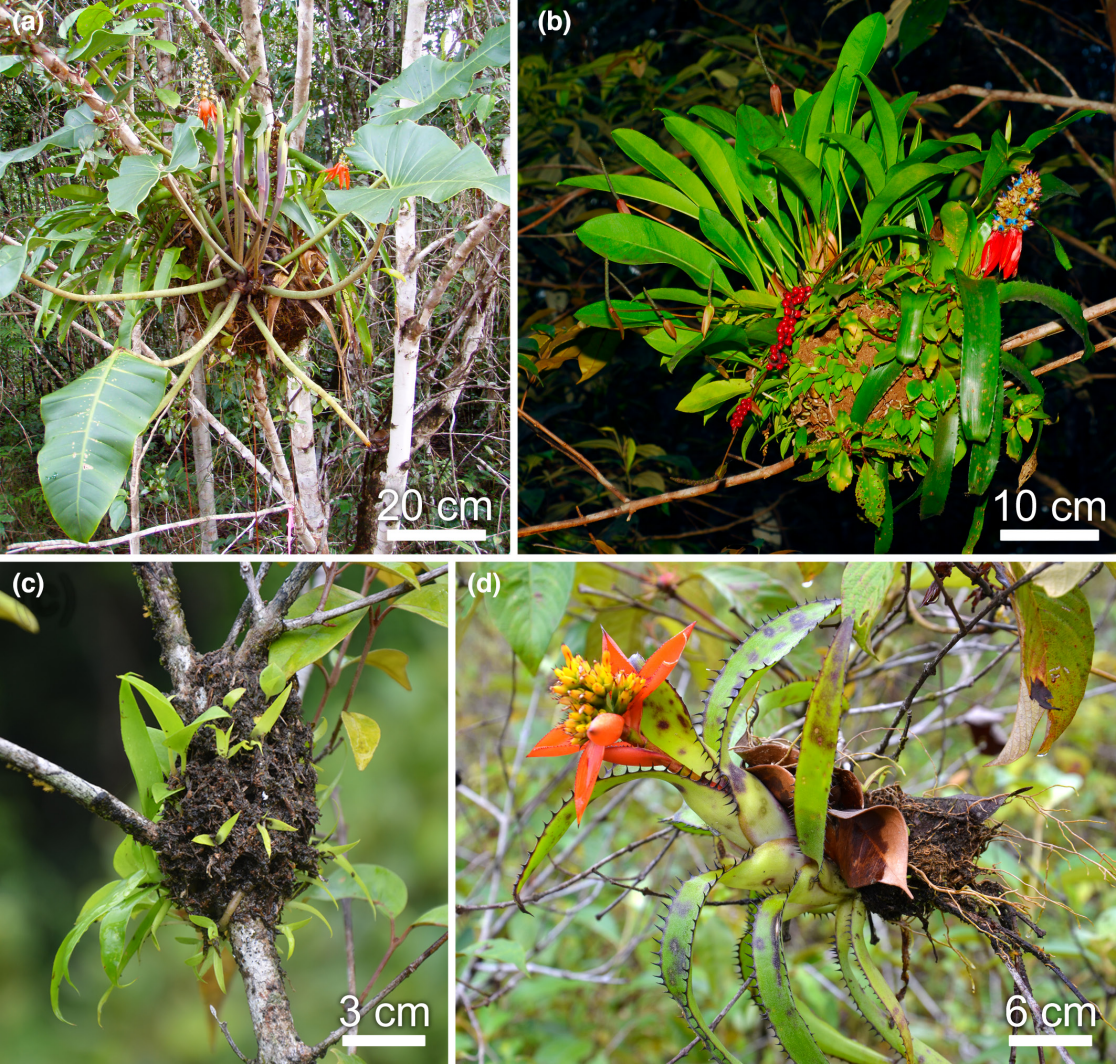
Two examples of ant gardens. (a) *Camponotus femoratus* build large ant gardens generally in the canopy of Neotropical forests; here, we see the ant carton and epiphytes including *Philodendron* and *Anthurium* (Araceae) and tank bromeliads *Aechmea mertensii*. (b) Another *Camponotus femoratus* ant garden with *Anthurium* bearing fruits, the carton nest is clearly visible (photos Alain Dejean). (c) Foundation of *Neoponera goeldii* showing the rough carton nest made by several queens and their first workers; the epiphytes have begun to grow on this nitrogen‐rich carton. (d) A *Neoponera goeldii* ant garden with an *Aechmea mertensii* (Bromeliaceae) in bloom; the carton made by the ants is clearly visible (photos Bruno Corbara).

Ant gardens are mutualistic interactions between limited numbers of arboreal ant and epiphyte species (hereafter AG ants and AG epiphytes). Each AG ant species selects the seeds of several AG epiphyte species in their surroundings based on the volatiles released by the seed coats and incorporate them into a nutrient‐rich carton nest built by the founding queens and workers. Different AG ants can select the same AG epiphyte. These seeds then germinate, and their seedlings develop producing a root system that anchors the entire structure to the supporting tree (Figure 3). Thus, ant gardens represent one of the most evolved ant‐plant associations because they benefit from seed dispersal in favorable sites, are provided with nutrients, and are protected from herbivorous insects. In turn, the associated ants benefit from the structural stability of their nest and receive food rewards (e.g., fruit pulp and extrafloral nectar; Corbara & Dejean, 1996; Orivel & Leroy, 2011).

The presence of well‐developed hyphae has been shown in Southeast Asian ant gardens, but their function remains unknown (Kaufmann & Maschwitz, 2006; Weissflog et al., 2017).

A study on the epiphytic tank bromeliad *Aechmea mertensii* specifically associated with Neotropical ant gardens showed the presence of mycorrhizal fungi of the Glomerales and Diversisporales that, thanks to their network of filaments among the roots, provide this plant with moisture and nutrients that it would not obtain otherwise (Leroy et al., 2022). Furthermore, endophytic fungi belonging to the Ascomycota were also noted as well as some Basidiomycota to a lesser degree. Among the Ascomycota, Hypocreales, Chaetothyriales, and Eurotiales were more represented in the ant gardens initiated by the ponerine *Neoponera mertensii* than in those built by the formicine *Camponotus femoratus*, the contrary being true for Capnodiales. Thus, the identity of the ant species influences the root‐associated fungal diversity, endophytic fungi having a beneficial effect on the host plants (Leroy et al., 2019, 2022). It would be interesting to verify in the future if the same is true for other ant garden epiphytes and if the endophytic fungi participate in reinforcing these ant gardens.

## 6 BACKGROUND INFORMATION ON ANTS USING FUNGI TO REINFORCE THEIR CONSTRUCTIONS

Farming for structural materials was shown for several ant species belonging to different subfamilies using the hyphae of filamentous Ascomycota mostly of the Chaetothyriales and the Capnodiales to reinforce their constructions (e.g., carton nests, runway galleries, and shelters to protect attended hemipterans), whereas strains of other fungi recorded in these constructions are likely contaminants coming from spores. Both the Chaetothyriales and the Capnodiales are referred to as “sooty molds” due to their similar appearance and ecological niche, but they belong to separate classes, the Eurotiomycetes and the Dothideomycetes, respectively (Vasse et al., 2017; Voglmayr et al., 2011).

During the growth of filamentous fungi, only the young, apical parts of the hyphae are alive and active. They leave behind the distal parts that gradually age and then die, forming very resistant tube‐shaped cell walls mainly composed of chitin, glucans, and glycoproteins (Gow et al., 2017; Heitman et al., 2018). Indeed, these fibers keep their structural properties, remaining sturdy after the death of the mycelium, so that they are being used to create new composite materials in the construction industry (Wagner et al., 2021; Yang et al., 2021).

## 7 FUNGAL HYPHAE REINFORCING ANT GALLERIES

**FIGURE 4:**
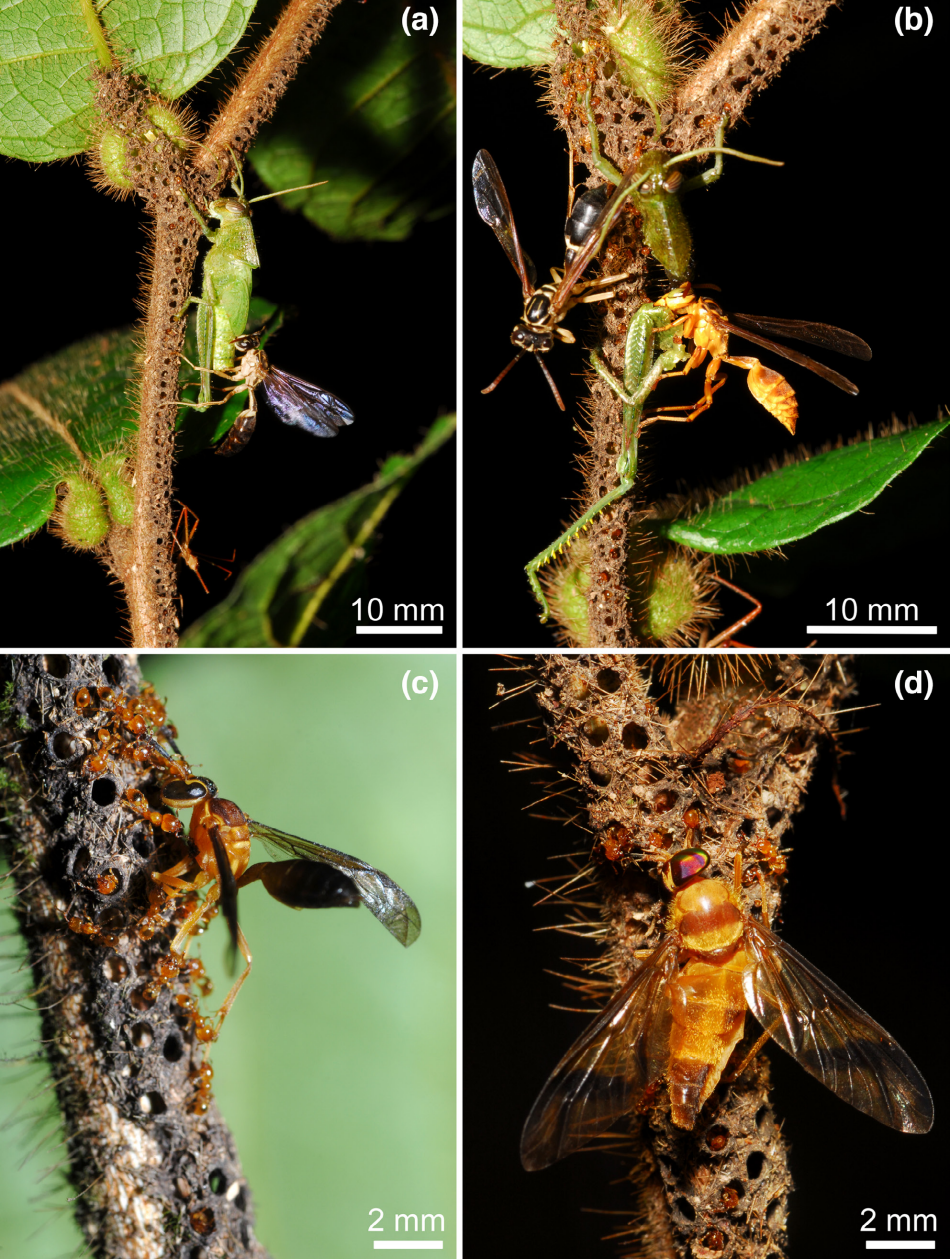
Gallery‐shaped traps built by *Allomerus decemarticulatus* on the myrmecophyte *Hirtella physophora*. The workers use intact host plant trichomes to form pillars onto which they build the vault of the galleries using cut trichomes reinforced by the hyphae of Chaetothyriales to form a composite material pierced by numerous holes. (a) The workers that ambush prey by placing themselves under the holes have captured a red reduviid bug and a grasshopper, whereas an *Agelaia cajennensis* wasp is robbing pieces of that prey (cleptobiosis). (b) Later, the *Agelaia cajennensis* was captured in turn, but an *Agelaia pallipes* successfully robbed several pieces of the grasshopper. (c) Small social wasps were easily captured. (d) Capture of a tabanid deer fly (photos Alain Dejean).

Workers of the plant‐ants *Allomerus decemarticulatus* and *Al. octoarticulatus* (Myrmicinae) associated, respectively, with the myrmecophytes *Hirtella physophora* (Chrysobalanaceae) and *Cordia nodosa* (Boraginaceae) build gallery‐shaped traps to capture different kinds of arthropods. These galleries are composed of intact host plant trichomes that act as pillars, whereas the vault of the galleries is shaped by severed trichomes reinforced by the hyphae of the Chaetothyriales. The whole forms a composite material pierced by numerous holes from which workers ambush prey (Dejean et al., 2005; Orivel et al., 2017; Ruiz‐González et al., 2011, 2019; Vogel, 2012; Figure 4). Furthermore, as do the ants with their wastes, these hyphae participate in host myrmecophyte nutrient provisioning (Leroy et al., 2011, 2017).

A comparable case of a gallery‐shaped trap is seen in *Azteca brevis* (Dolichoderinae) that builds along the stems of its host myrmecophyte *Tetrathylacium macrophyllum* (Salicaceae) runway galleries of a crusty black carton consisting of masticated plant material reinforced by Chaetothyrialean hyphae (Mayer & Voglmayr, 2009). Here, too, the workers hide under holes in the carton to ambush prey and these galleries can also be observed along the branches and trunks of trees of different species (Longino, 2007; Mayer et al., 2017; Schmidt & Dejean, 2018; Vogel, 2012).

## 8 FUNGAL HYPHAE REINFORCING SHELTERS FOR TENDED HEMIPTERANS

The workers of an African *Crematogaster* build carton shelters over hemipterans attended for their honeydew. This carton is composed of chewed wood and the fungal hyphae of Ascomycota likely belonging to the order Xylariales as the fungal fructifications were observed, permitting identification (Farquharson, 1914).

Another case of African *Crematogaster* building carton shelters to attend hemipterans was noted on the underside of the leaves of a *Macaranga* species (Euphorbiaceae). The carton of the walls consists of organic matter including tangled plant trichomes held together by the fungal hyphae of an Ascomycota likely belonging to the Chaetothyriales (i.e., very similar to those of *Hirtella*; Vogel, 2012).

## 9 FUNGAL HYPHAE REINFORCING THE CARTON OF NEST WALLS

Ants of the European genus *Lasius* (Formicinae) were the first noted as using a fungal mycelium to reinforce their nest walls (Fresenius, 1852). *Lasius fuliginosus* workers weed out competing molds and promote the growth of their carton fungi, supplying them with a mixture of honeydew and nectar (Elliott, 1915; Maschwitz & Hölldobler, 1970). Furthermore, as noted above, these fungi have a sanitary role as they secrete antibacterial compounds (Vasse et al., 2017). The hyphae, grown as nearly pure monocultures of Chaetothyriales and Capnodiales, are transmitted vertically (Schlick‐Steiner et al., 2008). Similarly, North American *Lasius* reinforce their nest carton with a fungal mycelium (Wild, 2010).

**FIGURE 5:**
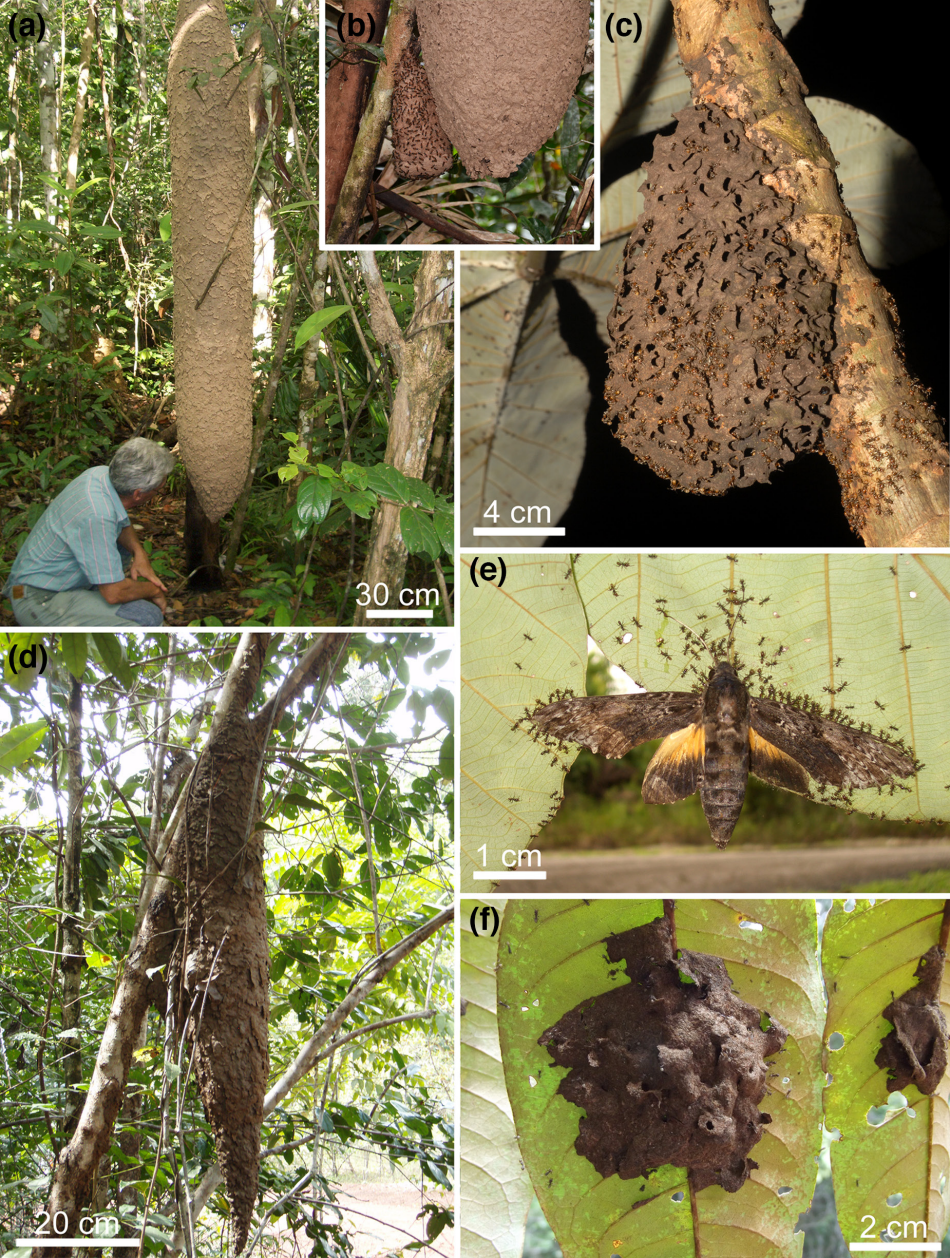
Brittle carton nest of dolichoderine ants reinforced by fungal mycelia of the Chaetothyriales. (a) Very large nest of the *Azteca* sp. *chartifex* group (b) The extremity of a similar nest with social wasps nesting side‐by‐side to be protected by the ants. (c) *Azteca andreae* build nests on the myrmecophyte *Cecropia obtusa*. (d) An *Azteca chartifex* nest. (e) *Azteca andreae* workers ambush side‐by‐side under *Cecropia obtusa* leaves permitting the capture of a large sphingid moth thanks to their hook‐like claws that catch on the fibrous loops on the undersides of the leaves recreating the Velcro® effect. (f) Nest of *Dolichoderus bidens* under a leaf (photos Alain Dejean).

Another case of fungal hyphae in the carton walls of ant nests is seen in the African arboreal myrmicine *Tetramorium aculeatum* whose workers build numerous nests under the leaves of their host trees or use several leaves to support their nests (Figure 5). The walls of these nests consist of silk, vegetal debris and fungal hyphae, the inside of the nest walls being lined with very fine vegetal debris and hyphae bearing fructifications (Bolton, 1980; Santschi, 1910; Wheeler, 1922).

**FIGURE 6:**
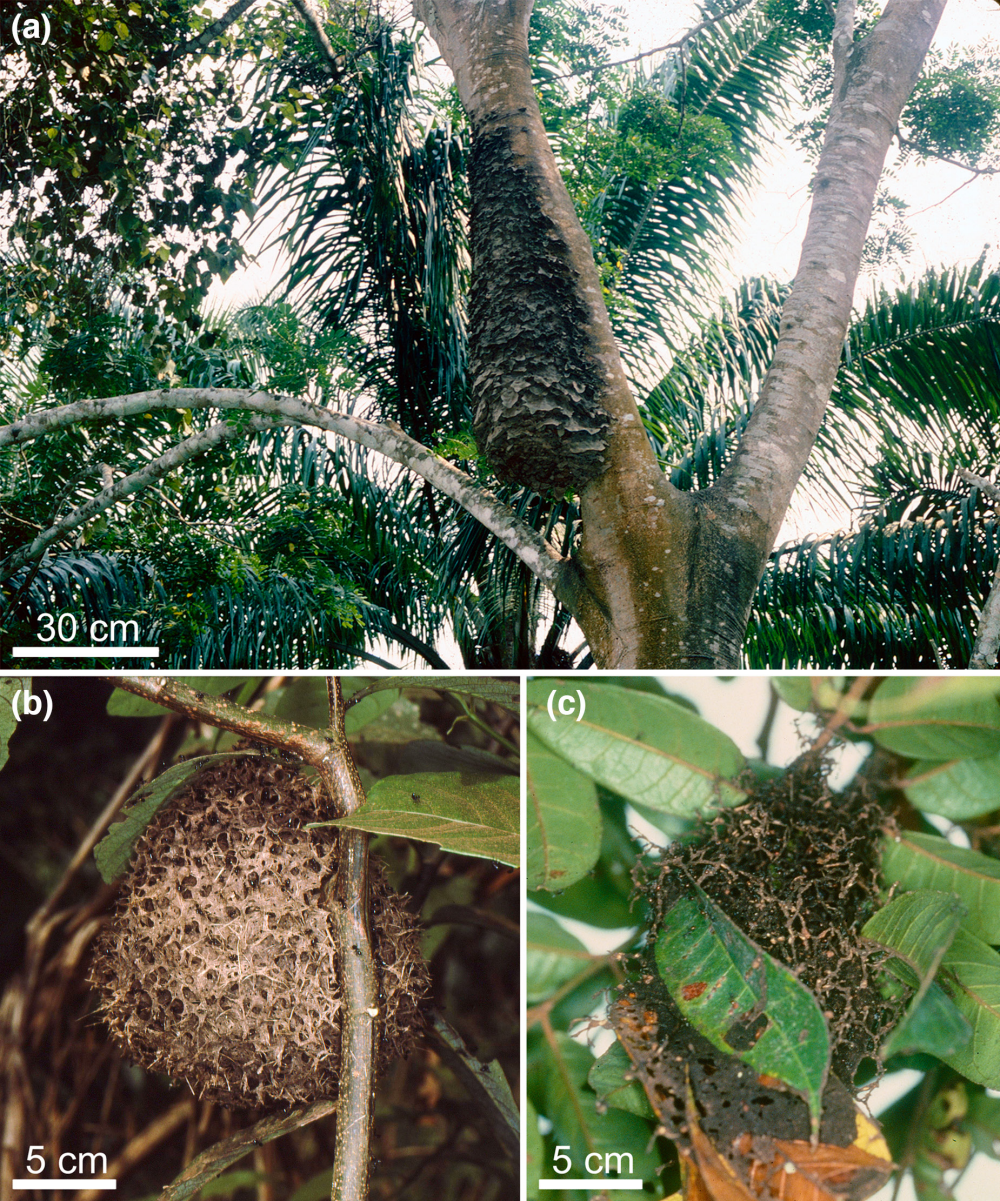
Brittle carton nest of *Crematogaster* ants also reinforced by Chaetothyriales fungi. (a) *Crematogaster stadelmanni* nest with large protrusions that likely permits heavy rain to run off (see Moumite et al., 2022). (b) Carton nest of *Crematogaster* sp. showing its external structure. (c) Nest of *Tetramorium aculeatum* made of carton between the leaves of the supporting tree (photos Alain Dejean).

Vasse et al. (2017) noted that carton‐building ants of the genera *Crematogaster* (Myrmicinae) and *Azteca* (Dolichoderinae) use fungal mycelia to reinforce the carton of their constructions. Only strains of Capnodiales and Chaetothyriales have a structural function in these composite materials (Figure 6). Among the *Crematogaster*, the nests of species belonging to the African subgenus *Atopogyne* using Capnodiales have an extremely hard carton with dense textures. The same is true for unidentified Malaysian *Crematogaster* and *Camponotus* species (Voglmayr et al., 2011; Taylor, 2015).

Species that use Chaetothyriales, including those from the genus *Azteca* and *Crematogaster* from subgenera other than *Atopogyne*, have brittle or friable carton nests (Quan et al., 2021, 2022; Voglmayr et al., 2011; see also Langshiang & Hajong, 2018).

## 10 BUILDING HARD CARTON NESTS AND LEAF‐CUTTING BEHAVIOR

Leaf‐cutting behavior in ants other than the higher Attina of the genera *Atta*, *Acromyrmex*, and *Amoimyrmex* has been reported only for the genus *Crematogaster* to the best of our knowledge, the first case being noted in Brazilian cocoa plantations where a nonidentified *Crematogaster* species acts as a defoliator (Urquhart, 1955). Yet, the description provided is not enough to prove whether there was true defoliation or whether this species was attacking leaf miners as is known for *Crematogaster gabonensis* in African palm tree plantations (Dejean et al., 1997). Among *Crematogaster* belonging to the subgenus *Atopogyne*, *Cr. buchneri* can skeletonize the leaves, buds, and flowers of 13 plant species including *Cola nitida* (Sterculiaceae; Eguagie, 1973), whereas *Cr. africana* workers attack cocoa trees, particularly the flowers (Taylor, 2015). Similarly, *Cr. wellmani* (former subgenus *Shaerocrema*) is another African arboreal carton‐nesting species that can defoliate Robusta coffee plants (Bruno de Miré, 1969). Yet, in these cases, the relationship between leaf‐cutting behavior and the use of fungi to reinforce their nests has not been examined or established.

**FIGURE 7:**
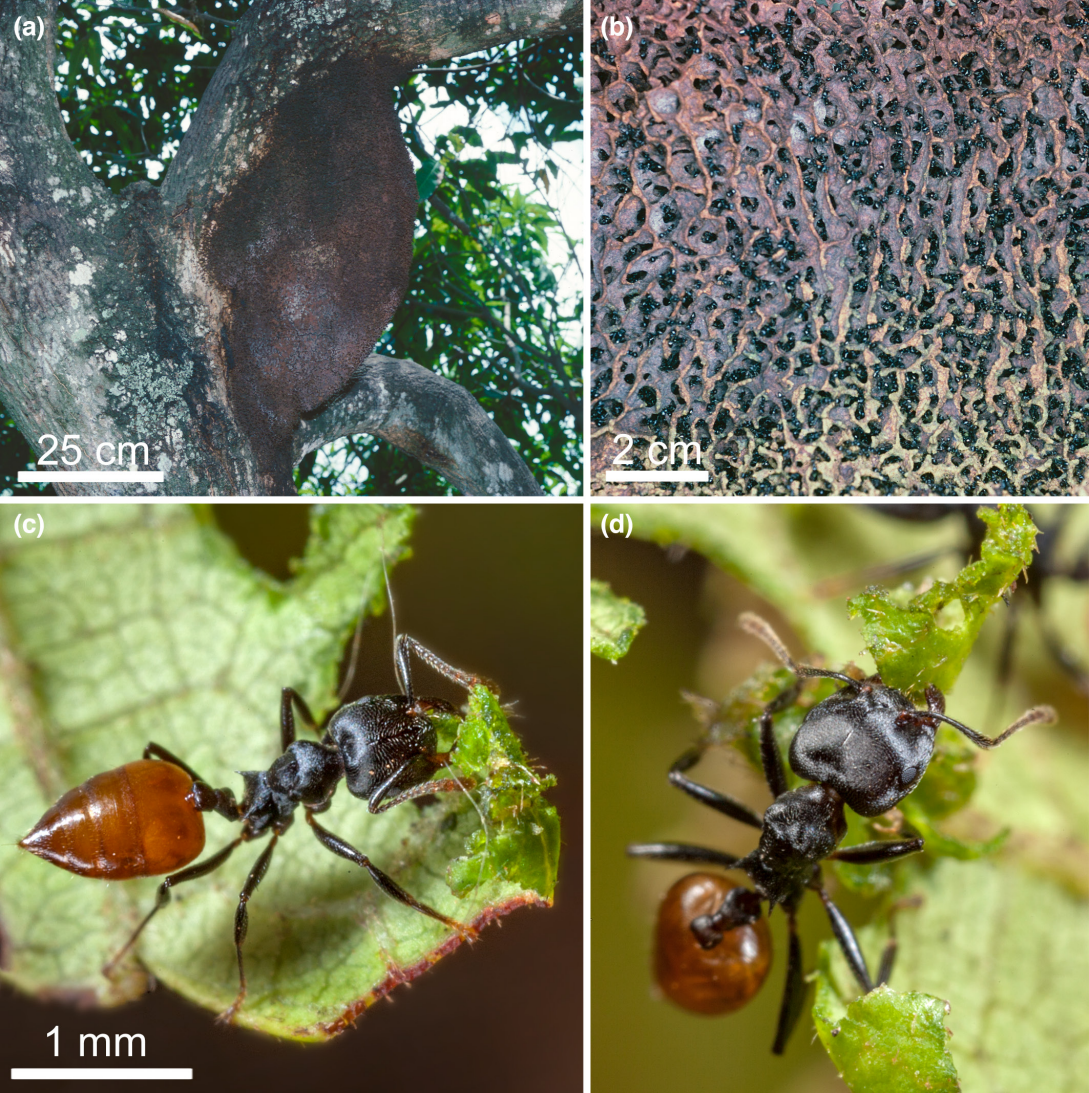
(a) Hard carton nest of *Crematogaster clariventris* reinforced by fungal mycelia of the Capnodiales. (b) Details of the hard carton inside the nest (photos Alain Dejean). (c, d) Workers cutting pieces of a young, nitrogen‐rich leaf (photos Piotr Naskrecki).

A case of convergent evolution with leaf‐cutting, fungus‐growing higher Attina is shown by *Crematogaster clariventris*, an Atopogyne that uses vegetal material to grow fungi (Figure 7). Yet, the similarities between leaf‐cutting Attina and *Cr. clariventris* stop there.

Indeed, there is a fundamental difference with leaf‐cutting Attina that cultivate Basidiomycota for food (the mycelium is kept alive), whereas *Cr. clariventris* workers grow fungi of the orders Capnodiales and likely Chaetothyriales to reinforce the carton of their nests (Dejean et al., 2023; Schultz, 2021; Figure 1). Because the mutualism is based on the death of the mycelium, there is a specific case of fungal agriculture for *Cr. clariventris* related to nest construction as noted for *Lasius* (Schlick‐Steiner et al., 2008). Here, the hyphae's cell walls remain sturdy, forming a natural composite material, something now widely used in the human construction industry (Wagner et al., 2021; Yang et al., 2021). Furthermore, the Attina belong to the New World and *Cr. clariventris* to the Old World and they belong to two distinct ant tribes of the subfamily Myrmicinae (Crematogastrini vs. Attini; estimated time of separation 70.8 Mya; origin of Myrmicinae 98.6 Mya; Ward et al., 2015). Finally, the mode of leaf cutting differs between the two taxa as *Crematogaster* workers have typical chewing mandibles that move symmetrically during defoliation (Paul, 2001) whereas leaf‐cutting Attina workers use their mandibles asymmetrically: The tip of one mandible grips the leaf surface, while the other mandible cuts through the leaf blade (Schultz, 2021).

## 11 CONCLUSIVE REMARKS

Ant‐fungus mutualisms involve ant species belonging to the subfamilies Dolichoderinae, Formicinae, Myrmicinae, and Pseudomyrmecinae. Among them, myrmicine species of the subtribe Attina that cultivate mostly Basidiomycota fungi of the family Agaricaceae for food present an ecological innovation with leafcutters using live plant parts to grow their associated fungi. Also, several plant‐ant species associated with phanerophytic or epiphytic myrmecophytes use mostly thin‐walled Ascomycota hyphae, many of them having antibacterial properties, whereas some Chaetothyriales provide food for the associated ant colonies, representing a convergence with the Attina. In addition, filamentous Chaetothyriales and Capnodiales form very resistant fibers that are used to reinforce ant constructions such as runway galleries, shelters to protect attended hemipterans, and carton nests. They form a natural composite material now widely used in the human construction industry.

During the course of evolution, ants have developed different types of interactions with other organisms extending from competition (lowers the fitness of the species involved), parasitism and predation (one species affects the other), commensalism (benefits one species, not the other), and mutualisms (benefit all species involved; Hölldobler & Wilson, 1994). Interpreted as central to the diversification of life, mutualisms can promote species persistence, ecological diversity, resistance to invaders and climatic changes (Dejean et al., 2022; Qian & Akçay, 2020; Thompson, 2006).

Although in most cases Attina transmit their garden fungi vertically, aligning the interest of both partners, horizontal transfer is possible in the lower Attina and for *Cyphomyrmex* involved in yeast agriculture. More generally, they are favored at the founding stage of the colonies, including in a leaf‐cutting species (Howe et al., 2019). In Attina, the mutualism is obligate as they depend on the fungi for food, while the fungi depend on the ants for survival (except for rare free‐living strains associated with lower Attina). Domestication is a multigenerational coevolutionary process between mutualist species where the domesticator constructs an environment favoring the survival and reproduction of the domesticated species (i.e., niche construction); in turn, the domesticator is provided with resources and/or services. This results in the evolution of traits that ensure the stable association of both interacting species across generations as their fitness is increased (Purugganan, 2022). Overall, fungi associated with lower Attina as well as yeast fungi were not domesticated and domestication is likely for coral fungi and demonstrated for fungi associated with higher Attina (Ješovnik & Schultz, 2022). Vertical transmission was also noted in the association between three plant‐ant species and their mutualist myrmecophytes that feed on Chaetothyrialean hyphae (Ascomycota), the same being true for the incipient colonies of *Azteca* spp. associated with myrmecophytic *Cecropia* (Blatrix et al., 2012; Mayer et al., 2018). Among ants using fungi to reinforce their constructions, vertical transmission was noted in *Lasius* that build their nest using fungal hyphae (Schlick‐Steiner et al., 2008).

Farming for fungi used for food, for their antibacterial properties, or to serve in construction was therefore noted, and it is probable that vertical transmission exists in many cases as this specificity is favored by the niche construction by the ants involved in these mutualistic relationships.

## AUTHOR CONTRIBUTIONS

**Alain Dejean:** Conceptualization (equal); writing – original draft (equal). **Frédéric Azémar:** Conceptualization (equal); resources (equal). **Piotr Naskrecki:** Resources (equal); validation (equal). **Maurice Tindo:** Conceptualization (equal); validation (equal); writing – original draft (equal). **Vivien Rossi:** Formal analysis (equal); validation (equal). **Christian Faucher:** Conceptualization (equal); visualization (equal). **Hervé Gryta:** Conceptualization (equal); validation (equal).

## CONFLICT OF INTEREST STATEMENT

The authors declare no conflicts of interest.

## Data Availability

Not applicable.
